# The Risk of Fractures in Patients with Type 1 Diabetes Mellitus

**DOI:** 10.3390/jcm15197468

**Published:** 2026-09-25

**Authors:** Emma Paulsson, Emilia Gauffin, Hans J. Arnqvist, Simona I. Chisalita

**Affiliations:** 1 Department of Health, Medicine and Caring Sciences, Faculty of Medicine and Health Sciences, Linköping University, 581 85 Linköping, Sweden; 2 Department of Endocrinology in Linköping, Region Östergötland, 581 85 Linköping, Sweden; 3 Department of Clinical Genetics, Linköping University Hospital, 581 85 Linköping, Sweden; 4 Department of Biomedical and Clinical Sciences, Linköping University, 581 85 Linkoping, Sweden

**Keywords:** type 1 diabetes, fracture, risk factors, women

## Abstract

**Background:** A variety of complications are inherent to type 1 diabetes mellitus, and some studies have shown an increased risk of fractures among patients with diabetes. Nevertheless, much about this phenomenon remains uncertain. Our aim is to determine if there is an increased risk of fractures among these patients compared to individuals without diabetes and to investigate the potential risk factors. **Methods:** This study was a population-based prospective observational study. Participants were included from 2010–2013 and all participants filled out a survey regarding risk factors. In 2022, approximately 10 years after inclusion, we gathered information about fracture events over this period. Cox regression was performed to determine whether there is an increased risk of fracture among type 1 diabetes patients, and risk factors for the complication were evaluated using the same statistical method. **Results:** An increased risk of fractures among type 1 diabetes patients compared to controls (HR 1.655, Cl 1.193–2.297, *p* = 0.003) was established. Women with type 1 diabetes had an increased risk compared to women without (HR 1.953, Cl 1.269–3.005, *p* = 0.002), whereas no significant differences were identified for men. Older age was a risk factor for both men and women with type 1 diabetes, as was diabetes duration for women. A higher Health Assessment Questionnaire (HAQ) score was associated with a greater fracture risk in both male and female patients with type 1 diabetes, as well as in male controls. **Conclusions:** Women with type 1 diabetes have a twofold greater risk of fractures compared to women without diabetes. Older age, higher HAQ score, and diabetes duration were identified as risk factors.

## 1. Introduction

Type 1 diabetes mellitus is a chronic disease that affects people globally, with consequences that affect a variety of systems within the body. For instance, the cardiovascular system is commonly affected with both micro- and macrovascular complications. Not as well-established are the consequences the disease has on an increased fracture susceptibility [1,2]. Previous studies have shown that type 1 diabetes patients are more likely to develop osteoporosis compared to controls [3,4,5]. A meta-analysis from 2015 determined that both men and women with type 1 diabetes have an increased risk of fractures compared to the general population. The authors of the study also concluded that larger prospective epidemiological studies are necessary to further verify these findings [6]. An increased number of fractures can lead to a general decline in a patient’s overall condition. This is especially true of older patients since their recovery time is much longer, and some degree of disability often remains after the fracture is fully healed. If connections between risk factors such as gender, age, BMI, etc., could be determined, our insight into the risk of fractures in type 1 diabetes would greatly improve [7]. Furthermore, primary prevention procedures (e.g., DXA analyses, physical activities, and dietary recommendations) could be implemented earlier and in accordance with the specific risk factor identified [8]. This is only the case, however, if the standardized primary prevention procedures are adequate for this particular patient group.

Several mechanisms and risk factors are potentially implicated in the pathology of fractures in patients with type 1 and 2 diabetes, one of which is bone turnover, i.e., the bone’s ability to create new bone [1]. Research has shown that individuals with type 1 diabetes have a lower BMD (bone mineral density), which increases the risk of fracture [1,9]. Furthermore, it is thought that individuals with both type 1 and 2 diabetes have an increased risk of fractures. A lower BMD is typically only seen with type 1 diabetes, and people with type 2 diabetes often have an increased BMD compared to the general population [3,5,9,10]. Low BMI increases the risk of low BMD in the femur and lumbar spine among patients with type 1 diabetes [11].

Poor glycemic control and longer disease duration have also been connected to low BMD and an increased risk of fractures [10,12,13,14]. Hyperglycemia is thought to influence bone development by means of osmotic damage and the suppression of genes connected to the maturation of osteoblasts [2].

It is thought that other complications in type 1 diabetes are also involved in the high risk of fractures among this patient group. Some studies have shown that retinopathy and peripheral neuropathy increase the risk of falls and, therefore, the risk of fractures among diabetes patients [2,7,12,13,15]. These two common complications also lead to a decrease in physical activity, which in itself is a risk factor for osteoporosis and fractures [15]. Micro- and macrovascular complications may also be associated with low BMD [15,16]; however, not all authors agree with this conclusion [14].

HAQ-DI (The Health Assessment Questionnaire Disability Index) is used to assess activity limitations. It consists of 20 questions concerning daily activities, and the final score is calculated as the mean of the category scores. To the best of our knowledge, no previous studies have connected the risk of fracture and HAQ scores for patients with diabetes. However, this instrument has been used in a number of studies to assess disability among type 1 and 2 diabetes patients [17].

Determining the risk factors for fractures is important because it would make it possible to predict which patients will develop fractures and what preventive action should be taken. The aim of this study is therefore to investigate whether patients with long-standing type 1 diabetes face an increased risk of fractures over a 10-year period compared to controls without diabetes. We also aim to determine whether any specific subgroups within the type 1 diabetes group face a particularly high risk and to identify risk factors that predict fractures.

## 2. Materials and Methods

### 2.1. Participants

The study population in this observational prospective investigation consists of the previously described population-based LeDIG cohort [18]. The study was conducted in cooperation with nine hospitals in southeastern Sweden between 2010 and 2013. The LeDIG cohort includes 1481 individuals, of whom 773 (350 men and 423 women) are patients with type 1 diabetes. The control group comprises 278 men and 430 women. All patients with type 1 diabetes experienced disease onset before 35 years of age, and had a minimum disease duration of 20 years. These patients were identified by local diabetes registries and the Swedish National Diabetes Registry (NDR), and were invited to participate in the study by mail, along with the relevant questionnaire. The control participants were selected from the Swedish general population using the Swedish national population register. They were matched for sex and age (±5 years) and invited to the study concomitant with the type 1 diabetes patients. Individuals with diabetes or with elevated fasting plasma glucose (levels ≥ 7.0 mmol/L) were excluded from the control group (17 individuals). If an invited control participant declined or did not respond, the next eligible matched control was contacted for recruitment. A very large majority of individuals with type 1 diabetes receive specialist diabetes care at hospital outpatient clinics, which in turn report to the NDR. Therefore, the NDR was considered a suitable route for recruiting individuals with type 1 diabetes. In total, 1727 type 1 diabetes patients in the NDR met the recruitment criteria and were contacted for recruitment; of these, 773 (45%) agreed to participate. Among the 1995 invited controls, 721 (36%) participated. A previous dropout analysis among individuals with type 1 diabetes showed that non-participants were younger and more often male than participants [18].

### 2.2. Data Collection

At recruitment, the patients filled out a questionnaire that collected information on various potential risk factors. The first part of the survey was identical for both groups; however, the questionnaire for diabetes patients included an additional part concerning aspects of their disease. Both background information and comorbidities were included in the survey. Information about laser treatment for retinopathy, weight, height, HAQ score, the year the patient was diagnosed, and smoking status was also collected. The participants’ age was derived from their Swedish social security number.

Data about fracture events was gathered from the patients’ recruitment to the study until the start of 2022. Data was extracted from the Swedish national in- and outpatient register, a very detailed and well-managed register that contains records of all individual diagnoses patients receive; we requested all relevant fracture diagnoses (S12, S22, S32, S52, S72, and S82). The selection intended to capture all fractures that could potentially be associated with underlying bone disease, while excluding fractures that were more likely to be attributable to specific trauma. For example, fractures of the hands and feet were not included, as these were considered to be more likely to result from specific traumatic events rather than underlying skeletal fragility. However, to ensure that recurrence did not affect our results, only the first fracture event was included in the statistical analyses. Diagnoses are shown as ICD-10 codes (International Classification of Diseases), along with the date the diagnosis determined by the patient’s physician. Data was not directly collected from patient journals. Instead, the date used is the date the physician established the diagnosis, which may not necessarily reflect the exact date when the patient sustained the fracture. Consequently, to ensure that only incident fractures were included, we included a fracture diagnosis only if it was the primary diagnosis.

### 2.3. Ethical Considerations

All participants provided written consent to participate in the study, and the study was approved by the Swedish ethics committee on 14 October 2003 (DNR 03-386), with renewed approval obtained on 15 February 2017 (DNR 2017/72-32). To access data from the Swedish national in- and outpatient register, another application was submitted and approved on the 13 November 2021 (DNR 39921/2021). The study protocol followed the principles expressed in the Declaration of Helsinki.

### 2.4. Statistical Methods

All statistical calculations were performed using IBM SPSS version 29. Descriptive statistics were conducted, including the number and percentage of individuals with fractures and the associated risk factors. For continuous variables, the median, Q1, and Q3 were calculated. Between-group differences for baseline characteristics were calculated using Mann–Whitney U tests for continuous variables and chi-square tests for categorical data. Cox regressions were performed to identify potential risk factors. Analyses were performed for the entire cohort, and also for subgroups stratified by gender and study group. Spearman correlations, chosen to minimize the effect of potential outliers and multicollinearity, were performed to evaluate potential connections between relevant risk factors. The associations between time to fracture and type 1 diabetes status in women and men were assessed using Kaplan–Meier analyses, and the significance of differences in fracture-free time was assessed using Mantel–Cox tests.

Statistical significance was set at the *p* < 0.05 level.

## 3. Results

### 3.1. Characteristics upon Inclusion: 2010–2013

Participants in the control group were slightly older compared to the diabetes patients (median age: 56 vs. 51 years, *p* < 0.001); within this group, there was also a significant age difference between men and women (median age: 58 vs. 55 years, *p* < 0.001). In the entire population there was a significant difference in age between men and women (median 55 vs. 53 years, *p* = 0.001). Patients with type 1 diabetes mellitus generally had higher HAQ scores than controls (median HAQ-score 0.000 vs. 0.000, *p* < 0.001). In both the patient group and the control group, women had higher HAQ scores than men (median HAQ-score: 0.140 vs. 0.000, *p* < 0.001 and median score: 0.000 vs. 0.000, *p* = 0.007, respectively. In the entire population there was a significant difference in age between men and women (median HAQ 0.000 vs. 0.000, *p* < 0.001). Within the control group the men have a significantly higher BMI (median BMI 26.30 vs. 24.85, *p* < 0.001), and in the entire population the men also have a significantly higher BMI (median 25.9 vs. 25.34, *p* = 0.005). Diabetes duration was significantly higher in women (median disease duration: 33 vs. 35, *p* = 0.039).

### 3.2. Fracture Events at Follow-Up: 2022

In total, 163 individuals experienced a fracture, 108 in the patient group and 55 among the controls (*p* = 0.001) (Figure 1, Table 1). Proportions of different types of fractures varied between patients and controls. The patient group experienced a significantly higher number of femur fractures (S72) (*p* < 0.001). There was no significant difference in the frequency of fractures of the lower arm (S52) between patients and controls (*p* = 0.070). Regarding this type of fracture, the incidence was higher for women than men within the patient group (*p* = 0.015, 40 vs. 17 fractures). Dropout that could impact the results occurred when participants died during follow-up prior to sustaining a fracture, precluding assessment of whether they would have experienced a fracture later. In total 16 control group participants died during the study (of whom 14 died before they sustained a fracture), as did 67 type 1 diabetes patients, of whom 58 had not sustained a fracture. In the patient group, there were significant age, diabetes duration, and HAQ score differences between those with fractures and those without (Table 1). In the control group, significant differences were observed between the same subgroups regarding smoking status and almost HAQ score (*p* = 0.070).

In examining the entire cohort using multivariable Cox regression, we found that type 1 diabetes was a significant risk factor (*p* = 0.002; HR 1.794; Cl 1.250–2.574) for fractures even when adjusting for other risk factors (Table 2). None of the other risk factors, except HAQ score (*p* = 0.002; HR 1.818; Cl 1.249–2.646) and age (*p* < 0.001; HR 1.044; Cl 1.024–1.064), were significant in this multiple regression, meaning that a higher HAQ score, age, and a diabetes diagnosis are risk factors for fractures even when adjusted for other common risk factors such as gender, smoking, and BMI.

Univariate regressions show that, compared to the control group, type 1 diabetes patients face an increased risk of developing fractures (HR 1.655; Cl 1.193–2.297; *p* = 0.003) (Table 3). In sex-stratified analysis, only the female diabetes population had a statistically significant increased risk of sustaining fractures compared to the control group (HR 1.953; Cl 1.269–3.005; *p* = 0.002). In the female population with type 1 diabetes, we found an increased risk of developing fractures in patients with a longer disease duration (HR 1.034; Cl 1.009–1.059; *p* = 0.007), a higher HAQ score (HR 2.080; Cl 1.322–3.270; *p* = 0.002), and older age (HR 1.066; Cl 1.036–1.098; *p* <0.001) (Table 3). For the male population with diabetes, older age (HR 1.039; Cl 1.003–1.075; *p* = 0.032), a higher HAQ score (HR 2.533; Cl 1.287–4.984; *p* = 0.007), and a higher BMI (HR 1.072; Cl 1.002–1.147; *p* = 0.043) were also significant risk factors (Table 3). There was also an association between the risk of fracture and a higher HAQ score in the male control group (HR 5.116; Cl 2.088–12.533; *p* < 0.001).

Using the same subgroups as shown in Table 3 (splitting the population based on gender and type 1 diabetes diagnosis), a multiple Cox regression was performed combining diabetes duration, HAQ, and age, and some significant associations were identified. In the subgroup of women with type 1 diabetes, age had a *p*-value of <0.001 (HR 1.066; Cl 1.028–1.106), which could be due to there being a significant correlation between age and diabetes duration (*p* < 0.001), both at the entire population (rho 0.564) and within both genders separately (men: rho 0.529; women: rho 0.607). In the subgroup of men without type 1 diabetes, there was also a significant association indicating that a higher HAQ value increased the risk of fracture (HR 2.342; Cl 1.182–4.642; *p* = 0.015).

In the female population, a Mantel–Cox-test in the Kaplan–Meier analysis yielded a significant *p*-value (*p* = 0.002). In contrast, the test for the male population did not produce a significant *p*-value (*p* = 0.283) (Figure 2a and Figure 2b, respectively).

## 4. Discussion

We identified an increased risk of developing fractures among type 1 diabetes patients, both in the entire population and when looking only at female participants. The risk factors that were deemed statistically significant for women with type 1 diabetes were age, HAQ score, and diabetes duration.

### 4.1. Fractures Among Patients with Type 1 Diabetes vs. The General Population

Our findings show that the risk of fractures is particularly high for women within the patient population, even to the extent that it increases the risk for the entire diabetic group compared to the control group. The fact that gender was not a significant risk factor in the entire population suggests that our conclusion that women with type 1 diabetes face a particularly high risk of fractures compared to men is correct. However, this finding is limited by the smaller number of men in our study, and a significant result may have been observed with a larger study population. Most previous research on the subject has looked at populations as a whole without stratifying analyses by gender [5,10]. Nevertheless, a meta-analysis from 2015 determined that both men and women with type 1 diabetes face an increased risk of fractures compared to the general population [6]. Moreover, a study from 2009 investigated BMD by comparing patients with type 1 diabetes to the general population, finding that there was no significant difference in BMD among women, but that men with type 1 diabetes had a lower BMD compared to the control group. It should be noted that the study was conducted on a small population of just 102 participants [14]. Another study conducted on a population consisting only of women found that women with type 1 diabetes face an increased risk of hip fractures compared to the control group. However, this study looked only at postmenopausal women [19]. We also found an increased risk of femur fractures among our type 1 diabetes patients compared to the control group.

In our study, fracture data were obtained in the form of ICD codes, which do not provide information on the mechanism of injury or whether a fracture was associated with high- or low-energy trauma. To minimize the risk of including fractures that resulted from major or specific trauma, we restricted the analysis to fracture sites that could plausibly be associated with low-energy trauma and underlying skeletal fragility. Thus, fractures more commonly associated with specific traumatic events, such as fractures of the hands and feet, were not included. This is of particular importance since we know little about what causes the increased susceptibility to fractures in patients with type 1 diabetes. Some studies suggest that individuals with type 1 diabetes may have reduced bone mass and BMD, which could contribute to an increased susceptibility to low-trauma fractures compared to individuals without type 1 diabetes [16]. However, it is not clear that this decrease in bone mass and density is the underlying issue; instead, the microarchitecture of the bones may play an important role. If we could be certain that solely low-impact fractures were included in the study, it might increase the odds of more specifically pinpointing the potential mechanism behind the occurrence.

### 4.2. Risk Factors

A significant risk factor identified in our patient population is age. Age being a risk factor is not surprising as it is known that senior individuals face an increased risk of osteoporosis and, in turn, fractures. For women, we found that a longer diabetes duration increased the risk of fractures, and the correlation between the two variables was found to be significant.

Our findings align with those of previous studies where a longer diabetes duration was found to be a risk factor for fractures in type 1 diabetes patients [12,14].

We do not conclude that BMI is correlated with an increased risk of fractures in the female group; however, in the male group with type 1 diabetes, a higher BMI did increase the risk of fractures. This finding conflicts with previous studies where a low BMI has been identified as a risk factor for a low BMD among people with type 1 diabetes [11]. We cannot be certain of the reasons for this. However, it has previously been established that the lower risk of fractures in patients with a higher BMI is connected to a higher BMD [20]. The mechanism behind an increased fracture risk in type 1 diabetes patients may, however, not be connected to BMD but to an alternative mechanism that remains unknown.

Furthermore, whether the patient was a smoker or not was not significantly associated with a risk of fracture. This is surprising as previous epidemiologic research has demonstrated that smoking increases the risk of osteoporosis and is commonly known to increase the risk of fractures [21]. However, we observed a significant difference pertaining to smoking status between individuals with and without fractures in the control group, with fractures being more prevalent among smokers. Nevertheless, the connection between smoking and fractures remains unclear, and further research is needed to draw a definitive conclusion. It is likely that too few of our participants smoked for us to find a significant connection. In a previous study conducted as part of the LeDIG project, regression was performed between controls and patients and a significant association was identified [22].

In contrast to previous research, we did not find retinopathy to be a significant risk factor for fractures in type 1 diabetes patients [2,7,12,13,15]. However, not all authors agree with this conclusion [14]. The fact that we did not find retinopathy to be connected to an increased risk of fractures could be due to the possibility that fractures may not be associated with poor glycemic control, as previously suggested in another study under the same project, where HbA1c was not connected to the risk of fractures [22].

We found that higher HAQ scores, reflecting a higher degree of disability and difficulty with daily tasks, were associated with an increased risk of fractures. To the best of our knowledge, no previous studies analyzed the relationship between HAQ score and fracture risk among type 1 diabetes patients. HAQ score could in the future be used as a tool to estimate a patient’s risk of fracture. Indeed, HAQ score can be used to predict the risk of vertebral fracture among patients with rheumatoid arthritis, where a higher HAQ score was associated with a higher risk of vertebral fracture [23].

### 4.3. Limitations

There are several limitations to our study. Because some of the data were based on a survey filled out by the participants themselves, there is a risk of recall bias. Furthermore, as this was an observational study, we can therefore not definitively establish causality. We can identify statistically significant associations between our variables but we cannot determine the underlying reason for these connections. Another limitation is that risk factors that can vary over time were only measured at recruitment; however, most show long-term stability, particularly in adults, making baseline measurements a reasonable approach to long-term risk assessment. Furthermore, many epidemiological studies rely on baseline measurements to predict long-term outcomes, which remains a generally accepted method.

Other factors, such as exposure to high-risk activities, could also affect the risk of fractures. Therefore, including only certain low-energy fractures would be preferable, but this was not possible as data showing how each participant sustained their fracture was not available. Furthermore, the inclusion of high-trauma fractures may introduce heterogeneity into fracture outcomes, as these fractures are more strongly influenced by the magnitude and circumstances of the trauma than by intrinsic skeletal properties. This could make it more difficult to identify associations between T1D-related alterations in bone quality or relationships between microarchitecture and fracture risk. Another limitation is the fact that our population consists of 1481 participants. This is a significant number, but stratifying the population according to gender and patients vs. controls resulted in relatively small groups. Including a larger number of participants in the study may yield more statistically significant connections, as well as solidifying our findings. Furthermore, although the high collinearity between age and diabetes duration could also affect the results, it does not necessarily mean that the association between the covariates and fracture risk is invalid; rather, it limits the ability to disentangle the individual contribution of age from that of diabetes duration. A strength of this study is the thorough characterization of patients in the LeDIG cohort as having type 1 diabetes [18].

## 5. Conclusions

We identified an increased risk of fractures among people with type 1 diabetes. This connection was largely driven by women with type 1 diabetes having an increased risk of fracture compared to women without type 1 diabetes; the same was not the case for men. We also found that HAQ score, diabetes duration, and age are risk factors for fractures in female patients with type 1 diabetes. Our findings suggest that only women with type 1 diabetes have an increased risk of fractures, which is of particular interest as very little, if any, research from this perspective has been conducted to date. If our findings could be confirmed by other studies, they would contribute to valuable guidance for clinicians implementing preventive measures for people with type 1 diabetes at risk of developing fractures.

## Figures and Tables

**Figure 1:**
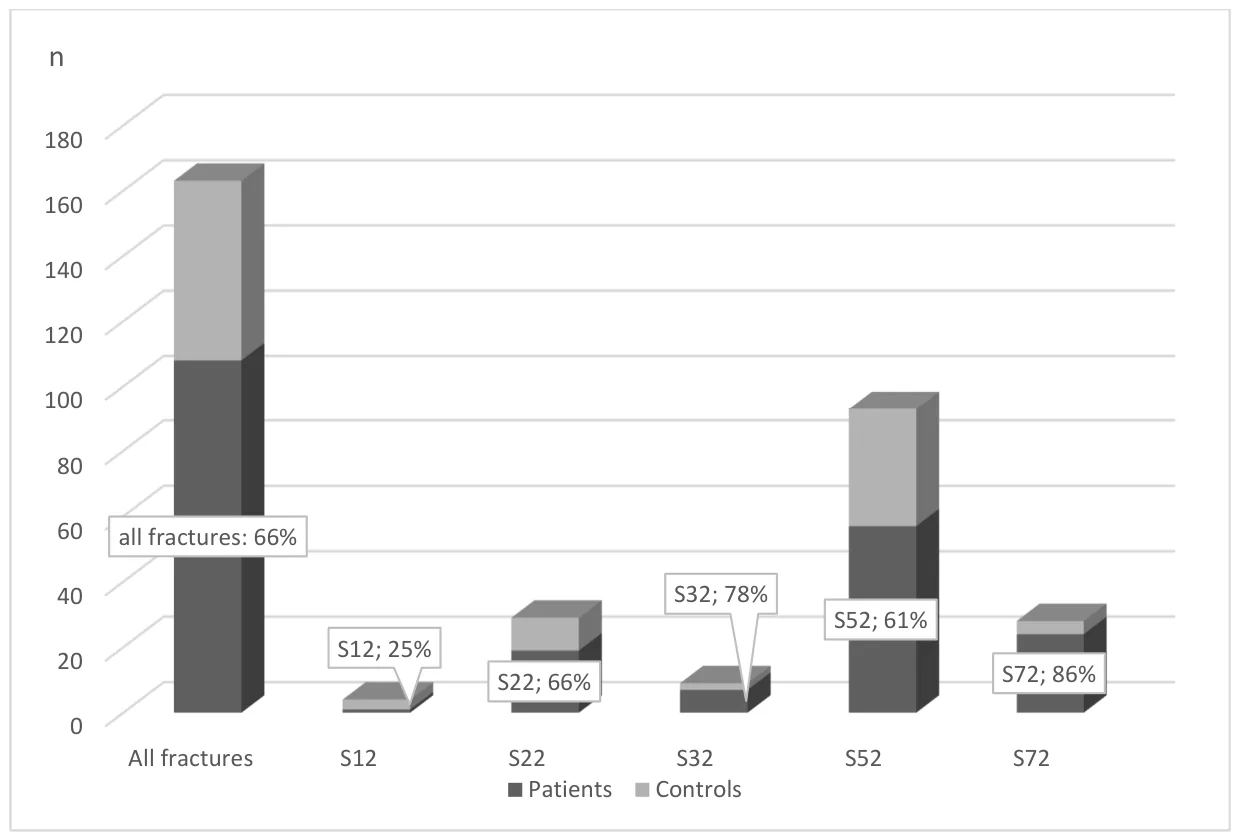
An overview of the types of fractures in our population. The *y*-axis shows the number of fractures; labels on the bars show the percentage of fractures in the patient group. S12: Fracture of a cervical vertebra; S22: Rib fracture; S32: Fracture of a lumbar vertebra; S52: Fracture of the lower arm; S72: Femur fracture.

**Figure 2:**
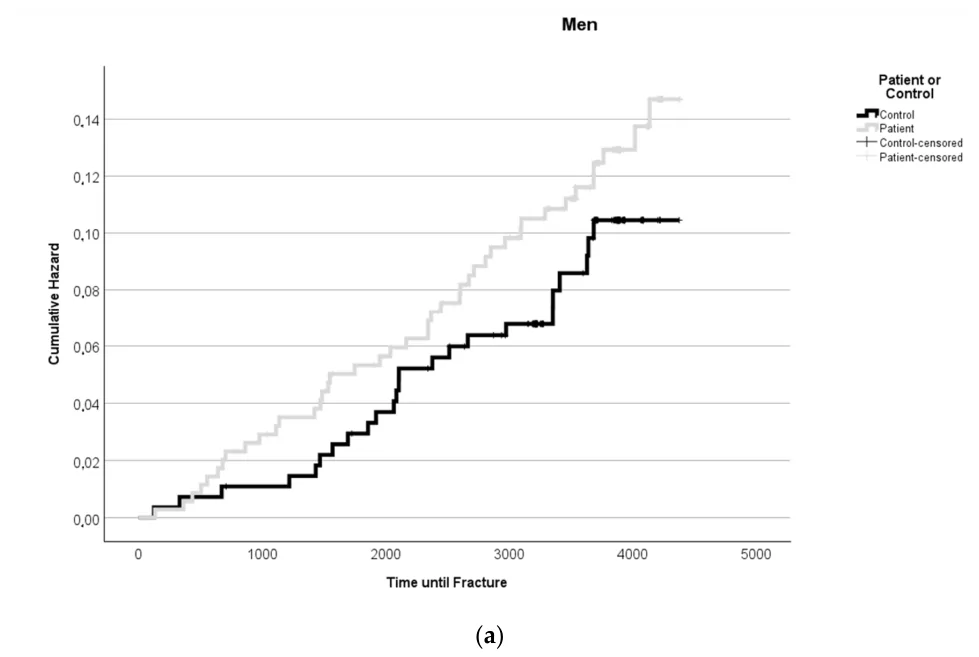

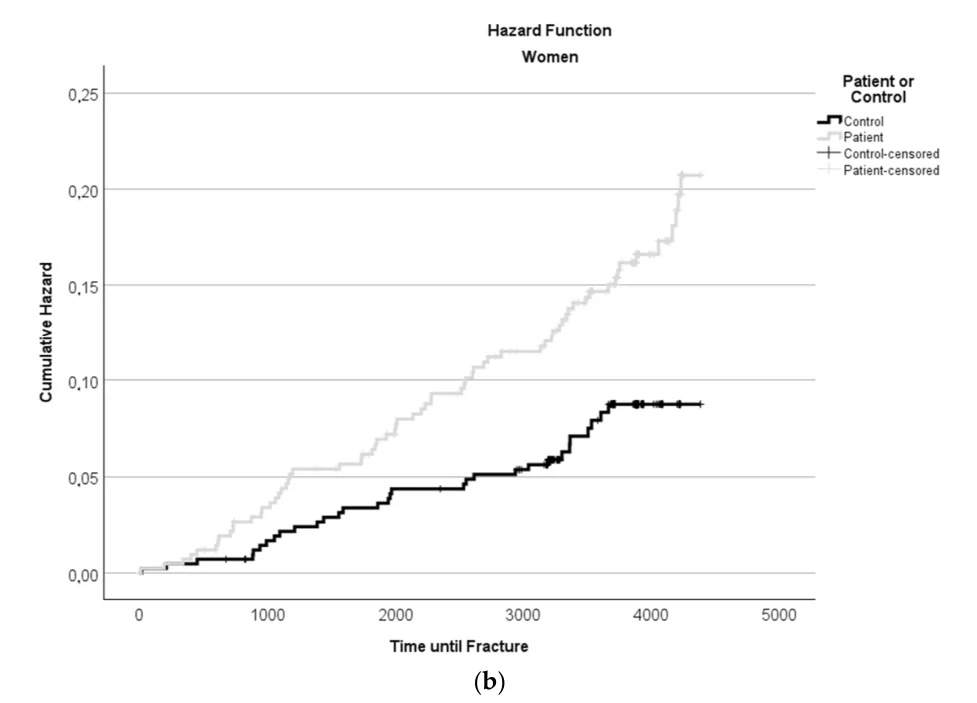
(**a**) Cumulative hazard function for the risk of developing fractures over time comparing patients and controls. A Kaplan–Meier analysis describing the risk of fracture over time, measured in days from the initiation of the study. No censoring was done in the analysis apart from the mandatory censoring within the analysis. Men only. (**b**) Cumulative hazard function for the risk of developing fractures over time, comparing patients and controls. A Kaplan–Meier analysis describing the risk of fracture over time, measured in days from the initiation of the study. No censoring was done in the analysis apart from the mandatory censoring within the analysis. Women only.

**Table 1:** Population characteristics in association with fracture events. The *p*-value was calculated between the groups using chi-square and Man–Whitney U tests. Diabetes duration and retinopathy were only calculated for participants with type 1 diabetes.

	Patients N (%)		*p*-Value	Controls N (%)		*p*-Value
	Fracture	No fracture		Fracture	No fracture	
**Number of individuals N**	108	665		55	653	
**Female N (%)**	66 (61)	357 (54)	NS	31 (56)	399 (61)	NS
**Smoker N (%)**	13 (12)	104 (16)	NS	14 (27)	90 (14)	0.010
**Age, median years (Q1, Q3)**	55 (49, 61)	50 (43, 58)	<0.001	57 (50, 64)	56 (47, 62)	NS
**Diabetes duration, median years (Q1, Q3)**	37 (29, 45)	33 (27, 41)	0.006	-	-	-
**BMI, median (Q1, Q3)**	26.14 (22.64, 28.34)	25.71 (23.51, 28.67)	NS	25.85 (23.66, 28.68)	25.42 (23.33, 28.02)	NS
**Retinopathy (%)**	48 (45)	253 (38)	NS	-	-	-
HAQ, median (Q1, Q3 *)	0.214 (0.00, 0.714)	0.00 (0.00, 0.429)	<0.001	0.00 (0.00, 0.179)	0.00 (0.00, 0.00)	0.070

Q1, Q3: 25th to 75th percentile; N: Number; NS: Not significant. Missing values: Gender 0, Smoking 23, Age 0, Diabetes duration 6, BMI 10, Retinopathy 3, HAQ 4. * Control group HAQ score: no fracture (min–max 0.00–2.00) vs. fracture (min–max 0.00–2.29). Patient group: no fracture (max–min 0.00–2.00) vs. fracture (max–min 0.00–2.14).

**Table 2:** Multivariable Cox regression analysis of potential risk factors for sustaining a fracture in the entire study cohort.

	HR	95% CI for HR		*p*-Value
		lower	upper	
**Smoking**	1.101	0.718	1.690	0.659
**Age**	**1.044**	**1.024**	**1.064**	**<0.001**
**BMI**	0.993	0.955	1.033	0.742
**HAQ**	**1.818**	**1.249**	**2.646**	**0.002**
**Patient versus control**	**1.794**	**1.250**	**2.574**	**0.002**
**Gender**	1.082	0.775	1.511	0.643

HR: Hazard ratio. The variables in bold marks significant associations.

**Table 3:** Several univariate Cox regression analyses of potential fracture-related risk factors. Each Cox regression was performed individually, and the file is split between both controls vs. patients and men vs. women. Because the controls did not have diabetes, regressions regarding diabetes duration and retinopathy were only performed for patients.

			N	HR	95% Cl for HR		*p*-Value
					lower	upper	
**Diabetes Diagnosis**							
	Total	**Patients and controls**	**1473**	**1.655**	**1.193**	**2.297**	**0.003**
	Men	**Patients and controls**	627	1.317	0.795	2.182	0.285
	Women	**Patients and controls**	**846**	**1.953**	**1.269**	**3.005**	**0.002**
**Smoking**							
	Men	**Controls**	270	2.097	0.827	5.318	0.119
		**Patients**	344	0.755	0.269	2.118	0.593
	Women	**Controls**	415	2.195	0.967	4.984	0.060
		**Patients**	419	0.756	0.374	1.527	0.435
**Diabetes Duration**							
	Men	**Patients**	346	1.017	0.985	1.049	0.298
	Women	**Patients**	**418**	**1.034**	**1.009**	**1.059**	**0.007**
**Retinopathy**							
	Men	**Patients**	347	1.391	0.754	2.568	0.291
	Women	**Patients**	421	1.379	0.846	2.247	0.197
**BMI**							
	Men	**Controls**	273	0.929	0.812	1.063	0.283
		**Patients**	**342**	**1.072**	**1.002**	**1.147**	**0.043**
	Women	**Controls**	416	1.064	0.985	1.149	0.113
		**Patients**	410	0.946	0.888	1.009	0.093
**HAQ**							
	Men	**Controls**	**275**	**5.116**	**2.088**	**12.533**	**<0.001**
		**Patients**	**346**	**2.533**	**1.287**	**4.984**	**0.007**
	Women	**Controls**	424	1.207	0.370	3.939	0.755
		**Patients**	**422**	**2.080**	**1.322**	**3.270**	**0.002**
**Age**							
	Men	**Controls**	277	1.022	0.970	1.076	0.414
		**Patients**	**348**	**1.039**	**1.003**	**1.075**	**0.032**
	Women	**Controls**	424	1.033	0.991	1.075	0.122
		**Patients**	**422**	**1.066**	**1.036**	**1.098**	**<0.001**

HR: Hazard ratio. The variables in bold marks significant associations.

## Data Availability

The original contributions presented in this study are included in the article. Further inquiries can be directed to the corresponding author.
